# Hereditary Hypercalcemia Caused by a Homozygous Pathogenic Variant in the* CYP24A1* Gene: A Case Report and Review of the Literature

**DOI:** 10.1155/2019/4982621

**Published:** 2019-04-08

**Authors:** Daniele Cappellani, Alessandro Brancatella, Martin Kaufmann, Angelo Minucci, Edda Vignali, Domenico Canale, Elisa De Paolis, Ettore Capoluongo, Filomena Cetani, Glenville Jones, Claudio Marcocci

**Affiliations:** ^1^Department of Clinical and Experimental Medicine, Unit of Endocrinology, University of Pisa, Pisa, Italy; ^2^Department of Biomedical and Molecular Sciences, Queen's University, Kingston, ON, Canada; ^3^Fondazione Policlinico Universitario A. Gemelli IRCCS, Roma, Italy; ^4^Università Cattolica del Sacro Cuore, Roma, Italy

## Abstract

**Introduction:**

Loss of function mutations of CYP24A1 gene, which is involved in vitamin D catabolism, cause vitamin D-mediated PTH-independent hypercalcemia. The phenotype varies from life-threatening forms in the infancy to milder forms in the adulthood.

**Case Presentation:**

We report a case of a 17-year-old woman with a history of nephrolithiasis, mild PTH-independent hypercalcemia (10,5mg/dL), and high serum 1,25(OH)_2_D concentrations (107pg/mL). Other causes of hypercalcemia associated with the above biochemical signature were excluded. Family history revealed nephrolithiasis in the sister. Blood testing in first-degree relatives showed serum PTH in the low-normal range and 1,25(OH)_2_D at the upper normal limit or slightly elevated. The CYP24A1 gene analysis revealed a known homozygous loss-of-function pathogenic variant (c.428_430delAAG, rs777676129, p.Glu143del). The panel of vitamin D metabolites evaluated by liquid chromatography showed the typical profile of CYP24A1 mutations, namely, low 24,25(OH)_2_D_3_, elevated 25(OH)D_3_:24,25(OH)_2_D_3_ ratio, and undetectable 1,24,25(OH)_3_D_3_. The parents and both the siblings harbored the same variant in heterozygosis. We decided for a watchful waiting approach and the patient remained clinically and biochemically stable over a 24-month followup.

**Conclusion:**

* CYP24A1* gene mutations should be considered in cases of PTH-independent hypercalcemia, once that more common causes (hypercalcemia of malignancy, granulomatous diseases, and vitamin D intoxication) have been ruled out.

## 1. Introduction

Pathogenic variants (PVs) in the human cytochrome P450 24 subfamily A member 1 (*CYP24A1*) gene are associated with Idiopathic Infantile Hypercalcemia (IIH, OMIM 143880), a rare disease recently related to vitamin D catabolism impairment [1]. The* CYP24A1* gene encodes a 24-hydroxylase enzyme, which catalyzes the degradation of the active form of vitamin D, 1,25-dihydroxyvitamin D [1,25(OH)_2_D] by multiple pathways [2].* CYP24A1* loss of function leads to an increase in serum 1,25(OH)_2_D concentration, which may be associated with various degrees of hypercalcemia and hypercalciuria and low-or-undetectable plasma parathyroid hormone (PTH) levels. The phenotype of IIH embraces a wide range of clinical scenarios [3], from severe forms diagnosed early in the infancy (severe hypercalcemia associated with dehydration, vomiting, nephrocalcinosis, and sometimes death) [4] to milder forms, often diagnosed in the adulthood during workout for recurrent nephrolithiasis [5]. Since the recognition in 2011[1] that PVs in* CYP24A1* are responsible for IIH, a large number of cases have been reported, leading to an increased insight into the diagnostic and therapeutic management of this disease [6, 7].

Herein we describe a case of recurrent nephrolithiasis and moderate PTH-independent hypercalcemia of undetermined origin referred to our outpatient clinic for further investigation. The familial nature of hypercalcemia prompted us to search for genetic causes and we identified a loss of function variant in the* CYP24A1* gene.

## 2. Case Presentation

A 17-year-old woman was referred to the Endocrine Unit of the University Hospital of Pisa for further evaluation of hypercalcemia associated with undetectable/low PTH levels.

Her clinical history was unremarkable except for a previous admission to the local Emergency Unit for renal colic 3 years before; an abdominal ultrasound revealed unilateral kidney stones. On that occasion, the patient was treated with analgesics and hydration and no further investigations were performed. One year later she underwent extracorporeal shockwave lithotripsy for the recurrence of renal colics. At that time, routine blood tests revealed hypercalcemia [12.4 mg/dL; (reference range 8.4-10.2)], hypercalciuria [390 mg/24h, (100-300)], and undetectable PTH (< 4 pg/mL; NV 8-40) and a 25-hydroxyvitamin D [25(OH)D) level of 37.4 ng/mL. The family history was unremarkable with the exception of nephrolithiasis in the sister.

At admission, physical examination was normal, with no evidence of major bone abnormalities. Lab tests confirmed hypercalcemia, hypercalciuria, and low/undetectable PTH levels; bone turnover markers were slightly above the upper limit of adult reference range (Table 1). Routine biochemistry was normal. Chest X-ray and abdominal and neck ultrasound were unremarkable. The long lasting hypercalcemia, the negative medical history beyond nephrolithiasis, and the normal imaging studies made unlikely the hypothesis of paraneoplastic hypercalcemia. Further evaluation revealed elevated serum levels of 1,25(OH)_2_D suggesting vitamin D-dependent hypercalcemia. A granulomatous disease could be ruled out on the basis of normal serum concentration of angiotensin converting enzyme and the absence of specific signs at chest X-rays.

**Figure 1:**
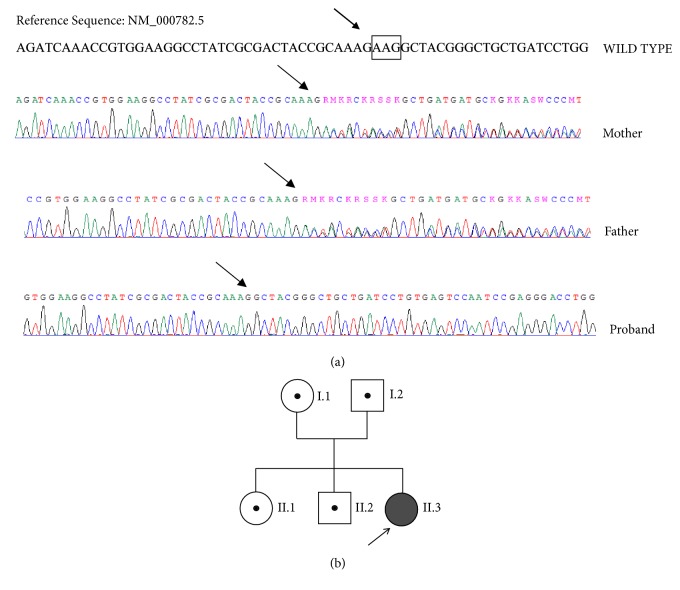
(a) Sequences of the* CYP24A1* exon 2 obtained by proband and her parents.* CYP24A1* gene amplification and sequencing were performed as reported. Sequences of the exon 2 obtained by proband and her parents were shown. Arrows indicate the position of* c.428_430delAAG* heterozygous and homozygous variant in the parents and the proband, respectively. (b) Family tree.

Because of the young age of the patient and the family history of nephrolithiasis, biochemical tests were performed in first-degree relatives. Total and ionized serum calcium, phosphate, PTH, and 1,25(OH)_2_D levels were in the normal range in both parents, who had a low vitamin D status. Interestingly, in the siblings PTH concentration was in the low-normal range and 1,25(OH)_2_D at the upper normal limit or slightly elevated (Table 2). The latter findings, together with the biochemical profile of the patient, suggested that hypercalcemia might be due to an impairment of the CYP24A1 catabolic pathway. The genetic analysis in the proband was made using High Resolution Melting Analysis (HRMA) [8] and further confirmed using gene amplification and sequencing [9], revealing a known homozygous PV (c.428_430delAAG, rs777676129, p.Glu143del) in the* CYP24A1* gene (Figure 1(a)). The same heterozygous variant was detected in the parents and the siblings (Figure 1(b)). The parents excluded consanguinity, even though they came from the same small village.

**Figure 2:**
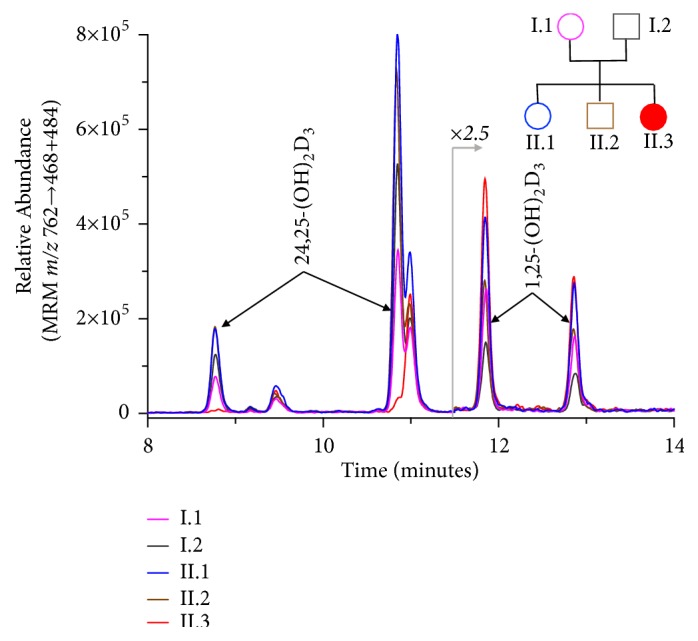
LC-MS/MS chromatogram of dihydroxylated vitamin D metabolites. Serum samples were prepared by immunoextraction and derivatized with DMEQ-TAD. Peaks corresponding to 6S and 6R isomers of DMEQ-TAD adducts of 24,25(OH)_2_D_3_ and 1,25(OH)_2_D_3_ were observed using the multiple reaction monitoring (MRM) transition of mass/charge (m/z) 762.3-* *->468.1+484.1, from which serum concentrations of these metabolites were determined. The figure reveals the dramatically reduced concentration of 24,25(OH)_2_D_3_ in the proband II.3 as well as elevated 1,25(OH)_2_D_3_, in comparison to unaffected family members.

To complete the biochemical profile of vitamin D metabolites, liquid chromatography tandem mass spectrometry (LC-MS/MS) was run on stored serum samples of all the family members. Serum samples were prepared by immunoextraction and derivatized with 4-[2-(6,7-dimethoxy-4-methyl-3,4-dihydroquinoxalinyl)ehtyl]-1,2,4-triazoline-3,5-dione (DMEQ-TAD), as reported [10]. We observed that the proband exhibited low 24,25(OH)_2_D_3_ (0.42 ng/mL) and elevated 25(OH)D_3_:24,25(OH)_2_D_3_ ratio (118; cutoff >80) which confirmed the diagnosis of impaired CYP24A1 function. A more rigorous chromatographic method[11] was also used to assay the same sample (25(OH)D_3_:24,25(OH)_2_D_3_ ratio = 3117; cutoff>140), which also indicated inappropriately low levels of 24,25(OH)_2_D_3_ in the proband. The other family members, who present as heterozygous variants, exhibited essentially normal serum 24,25(OH)_2_D_3_ concentrations and 25(OH)D_3_:24,25(OH)_2_D_3_ ratios (Table 3 and Figure 2).

Because of the mild hypercalcemia, we did not advise pharmacologic treatments aimed at modulating 1,25(OH)_2_D metabolism and we recommended maintenance of adequate hydration and avoidance of unprotected excessive sunlight exposure. Followup evaluation up to 24 months showed that the patient was in an overall stable condition, with serum calcium concentration slightly above the upper normal limit and renal ultrasound showing no recurrent nephrolithiasis.

The patient and the family gave written informed consent for the genetic analysis and the use of their clinical data for scientific purposes, including publication.

## 3. Discussion

Hypercalcemia is a common disorder, with a prevalence of 1/500 patient in the outpatient setting[12]. Primary hyperparathyroidism is the most common cause of hypercalcemia [13]. Vitamin D-induced hypercalcemia is a heterogeneous group of diseases that includes vitamin D intoxication, granulomatous diseases, and abnormalities of vitamin D metabolism. Hypercalcemia due to loss of function variants in the* CYP24A1* gene is a genetic disorder recently described in patients with IIH [1]. Nowadays the name “idiopathic infantile hypercalcemia” is considered a misnomer [6], because in most patients a genetic cause can be identified (namely, a loss-of-function mutation in the* CYP24A1* or in the* SCL34A1* or large deletions on chromosome 7 causing the Williams-Beuren syndrome), and the clinical phenotype is no longer confined to infancy.

Vitamin D is mainly produced in the skin or supplied by dietary sources. It undergoes an initial activation by 25-hydroxylation in the liver, catalyzed by CYP2R1, thus generating 25(OH)D. A second hydroxylation by 1*α*-hydroxylase (CYP27B1) takes place mainly in the kidney, but also in several extrarenal tissues, and converts 25(OH)D to 1,25(OH)_2_D, the active form of vitamin D [14]. The* CYP24A1* gene, located at 20q13.2, encodes the cytochrome P450 component of the mitochondrial 24-hydroxylase enzyme, which catalyzes the degradation of 25(OH)D and 1,25(OH)_2_D into the multistep 24-oxidation pathway to calcitroic acid [15, 16]. The* CYP24A1* gene has been cloned in animals [17, 18] and humans [19]. Its expression is induced by vitamin D receptor agonists [20] by interacting with a vitamin D response element in the promoter of the gene [18]. Furthermore, many hormones involved in bone mineral metabolism regulate the CYP24A1 enzyme. PTH attenuates the 1,25(OH)_2_D-mediated induction of the* CYP24A1* gene, through a direct effect on the transcription of the gene [21]. Fibroblast growth factor 23 (FGF23) decreases 1,25(OH)_2_D levels by inhibiting* CYP27B1* expression and inducing* CYP24A1* in the kidney [22].

*CYP24A1* loss-of-function variants are recognized as a cause of vitamin D-mediated hypercalcemia [23]. As a matter of fact, defective 24-hydroxylase activity results in high 1,25(OH)_2_D concentrations and, as a consequence, PTH-independent hypercalcemia with hypercalciuria, in the absence of hypophosphatemia. Twenty-one PVs of* CYP24A1* have so far been described in literature [6]. The disease is inherited as a recessive trait and a genotype-phenotype correlation has been postulated [3].

Biallelic variants, independently of whether in homozygosis or compound heterozygosis, result in a significant phenotype [1, 24], which may range from severe to mild and misrecognized forms [1, 3]. The majority of cases diagnosed in early infancy presents the classic manifestations of IIH, namely, severe hypercalcemia, dehydration, polyuria, vomiting, failure to thrive, nephrocalcinosis, muscular hypotonia, and lethargy, occasionally leading to death [1, 4, 23]. Conversely, cases with biallelic mutations diagnosed in the adulthood commonly present mild to moderate hypercalcemia [23] and recurrent nephrolithiasis [5, 25].

It is still not clear whether different PVs may be associated with different phenotypes. As a matter of fact, the specific PV may influence the extent of the variation in the enzyme activity, thus contributing to the severity of the clinical picture [26]. Specifically, most patients harboring p.Glu143del biallelic mutation present a late-onset clinical picture, which mainly consists in urological manifestations, such as nephrolithiasis and/or nephrocalcinosis [26–29]. Until now, the small number of patients so far reported does not allow drawing significant genotype-phenotype correlations both for the p.Glu143del and for other rarer PVs.

Data about heterozygote carriers are mainly derived from studies involving relatives of index cases carrying biallelic variants. Whether the presence of monoallelic mutation can lead to an overt clinical phenotype is still a matter of debate [24]. Heterozygote carriers usually have a milder biochemical phenotype compared to patients affected by biallelic variants [7, 25, 30, 31], with mild hypercalcemia and less frequently nephrolithiasis [25]. Moreover, others suggest that these patients are mainly asymptomatic and that incidental nephrolithiasis may be due to other causes [7]. This is in keeping with the finding in our kindred, where the clinical and biochemical picture in heterozygous mutation carriers was heterogeneous, thus suggesting that other factors might contribute to the phenotype (see below). Conversely, a study reported two children with monoallelic* CYP24A1* intron-exon splice junction mutations (IVS5+1G>A and IVS6-2A>G) with severe hypercalcemia and the classical phenotype of IIH, commonly due to biallelic mutation [31]. The authors postulated that the symptomatic picture could be due to an autosomal dominant inheritance pattern. Additional environmental factors or predisposing conditions, including vitamin D administration [1, 32], sunlight exposure [33], and pregnancy [34], may contribute to the development of a clinically relevant phenotype in patients with either biallelic or monoallelic mutations.

In clinical practice loss-of-function mutations in* CYP24A1 *should be searched in patients with hypercalcemia and hypercalciuria, associated with low serum PTH concentrations and 1,25(OH)_2_D levels in the upper normal range or slightly above. Serum 25(OH)D concentrations can be low, normal, or mildly elevated. The finding of markedly elevated 25(OH)D levels raises the suspicion of vitamin D intoxication. An additional diagnostic clue is the measurement of 24,25(OH)_2_D_3_, the main product of CYP24A1 [10], that is expected to be decreased. Unfortunately, the assay of this metabolite is not routinely available. Moreover, measurement of absolute 24,25(OH)_2_D_3_ concentration alone has limited diagnostic value, because low 24,25(OH)_2_D_3_ can also occur due to low 25(OH)D_3_ in addition to CYP24A1 mutation. We observed in patient I.1 (hetorozygote carrier) a 24,25-(OH)_2_D_3_ concentration of 0.56 ng/mL, similar to patient II.3 (proband). Calculation of a 25(OH)D_3_:24,25(OH)_2_D_3_ ratio indicates when 24,25-(OH)_2_D_3_ concentration is inappropriately low for a given 25(OH)D_3_ level. In unaffected individuals serum levels of 24,25(OH)_2_D are proportional to those of 25(OH)D and the ratio ranges between 5 and 25. In the patients affected by* CYP24A1* loss-of-function mutations, the ratio is markedly increased, up to more than 80 [10, 25, 31] by short method and over 140 by the long method, reported here. Currently, the measurement of the 25(OH)D:24,25(OH)_2_D is considered the most accurate screening tool for the identification of patients to be submitted to genetic testing.

Treatment of patient with* CYP24A1* PVs is directed towards the control of hypercalcemia. In severe cases, treatment starts with vigorous fluids administration eventually followed by a loop diuretic as furosemide when the patient is adequately hydrated. Other options include calcitonin and bisphosphonates. The use of corticosteroid to reduce intestinal calcium absorption is not advised in the setting of hypercalcemia related to* CYP24A1* PVs [35] because its therapeutic benefit requires a functioning CYP24A1 enzyme [36]. Another therapeutic approach aims to modulate the metabolism of 1,25(OH)_2_D. Ketoconazole reduces the synthesis of 1,25(OH)_2_D by inhibiting the CYP27B1 enzyme and has been effective in patients affected by* CYP24A1* loss-of-function mutations in the acute and in the chronic setting [31, 37]. Fluconazole has been proposed as a valid alternative to ketoconazole, especially for the less pronounced long-term toxicity [38].

Rifampin, given its capacity to induce CYP3A4 enzyme, catalyzes a nonspecific hydroxylation of 1,25(OH)_2_D to an inactive metabolite, 1,23,25(OH)_3_D, and has been used with overall good results [39].

Independently of the pharmacologic approaches, it seems reasonable to avoid exogenous vitamin D supplementation, implement a low-calcium diet, and avoid unprotected excessive sunlight exposure, even though the benefit of these approaches remains to be clarified [6].

In the case of females of child-bearing age with biallelic CYP24A1 mutations, it should be noted that the advent of pregnancy constitutes an added risk for hypercalcemia, as the placenta is a known site of additional 1,25(OH)_2_D_3_ synthesis. Several cases have been described in which the patient's hypercalcemia is exacerbated during recurrent pregnancies, a condition which dissipates during nonpregnant periods[28, 34].

## 4. Conclusion

The patient reported herein represents a typical case of homozygous* CYP24A1* loss-of-function mutation discovered in the early adulthood with recurrent nephrolithiasis.

About 2 years passed from the initial episode of renal colic to the first measurement of serum calcium and the discovery of hypercalcemia. This is a common finding in many adult patients presenting with nephrolithiasis and hypercalcemia due to PV of the CYP24A1 enzyme [1, 5, 38] and reflects the common attitude to approach the treatment of kidney stones rather than investigating the causes.

The differential diagnosis of hypercalcemia encompasses many different conditions. The possibility of mutation of the* CYP24A1* gene as a cause of hypercalcemia should be considered in cases of PTH-independent hypercalcemia, once that more common causes, namely, hypercalcemia of malignancy, granulomatous diseases, activated vitamin D intoxication, have been ruled out.

The clinical expression of the* CYP24A1* mutation is heterogeneous both in biallelic (age at diagnosis and severity) and monoallelic members of the same kindred (as in our family). Treatment should be individually tailored, taking into account the risk-benefit ratio. The severity of the clinical manifestations, the patient's age, the expected side effects of the medication proposed (which should be taken possibly for a lifetime), and the patient's preference should be taken into account.

## Figures and Tables

**Table 1:** Clinical and biochemical data at admission to our clinic.

Analyte	Result	Normal adult reference range
Total calcium (mg/dL)	10.5	8.6-10.2
Ionized calcium (mmol/L)	1.35-1.36-1.45*∗*	1.13-1.32
Phospate (mg/dL)	3.1	2.7-4.5
Magnesium (mg/dL)	2.02	1.7-2.2
Albumin (g/dL)	4.6	3.6-5.2
PTH (pg/mL)	< 4-7*∗*	8-40
Calcitonin (pg/mL)	< 2	< 11.5
25-hydroxy vitamin D (ng/mL) ^§^	30.3	
1,25-dihydroxyvitamin D (pg/mL) ^∫ ^	107	20-67
Osteocalcin (ng/mL)	61.6	6.8-34
Bone-specific alcaline phophatase (mcg/L)	23	2-20
Carboxy-terminal collagen crosslinks (ng/mL)	1.042	0.112-0.738
Urine calcium (mg/24h)	150-410-455*∗*	100-321
Urine phosphates (mg/24h)	697-875*∗*	400-1300
Urine magnesium (mg/24h)	140-164*∗*	60-120
Creatinine (mg/dL)	0.69-0.72*∗*	0.5-0.9
Urine creatinine (mg/24h)	1050-1085-1148*∗*	740-1570
Angiotensin converting enzyme (U/L)	80	65.8-114.4

*∗*When available, repeated measures are reported

§: 25(OH)D was assayed at University of Pisa laboratory as total 25(OH)D (i.e. the sum of 25(OH)D_2_ + 25(OH)D_3_) using a chemiluminescence immunoassay (IDS-iSYS, Immunodiagnostic systems, Boldon, Tyne and Wear, UK)

*∫*: 1,25(OH)_2_D was assayed at University of Pisa laboratory as total 1,25(OH)_2_D (i.e. the sum of 1,25(OH)_2_D_2_ + 1,25(OH)_2_D_3_) using a radioimmunoassay (IDS, Immunodiagnostic systems, Boldon, Tyne and Wear, UK)

**Table 2:** Clinical and biochemical findings in the patient's first degree family members.

	I.1	I.2	II.1	II.2
Age (years)	52	53	26	20
History of nephrolithiasis	No	No	Yes	No
Total calcium (mg/dL)	10	9.8	9.9	9.8
Ionized calcium (mmol/L)	1.28	1.23	1.29	1.26
Phosphate (mg/dL)	3.6	2.9	3.9	3.1
PTH (pg/mL)	17	18	11	8
25 hydroxyvitamin D (ng/mL)	7.8	17.3	28.4	22.5
1,25- dihydroxyvitamin D (pg/mL)	39	37	72	66

The reported values are the mean of two independent samples collected in two consecutive days. For family member identification see Figure 1(b). See Table 1 for the normal adult reference range at our laboratory and details about 25(OH)D and 1,25(OH)_2_D assays.

**Table 3:** Liquid chromatography tandem mass spectrometry analysis results.

	I.1	I.2	II.1	II.2	II.3
25(OH)D_3_ (ng/mL)	13.16	28.88	38.03	28.25	49.87
24,25(OH)_2_D_3_ (ng/mL)	0.44	1.14	1.94	1.66	0.02
25(OH)D_3_:24,25(OH)_2_D_3_ ratio	30.1	25.4	19.6	17.1	3117
1,25(OH)_2_D_3_ (pg/mL)	41.1	37.4	66.9	66.6	118.4
1,24,25(OH)_3_D_3_ (pg/mL)	6.5	7.7	16.9	21.4	< 2
